# Secreted protein acidic and rich in cysteine mediates active targeting of human serum albumin in U87MG xenograft mouse models

**DOI:** 10.7150/thno.34883

**Published:** 2019-10-11

**Authors:** Cho Rong Park, Jung Hwan Jo, Myung Geun Song, Ji Yong Park, Young-Hwa Kim, Hyewon Youn, Sun Ha Paek, June-Key Chung, Jae Min Jeong, Yun-Sang Lee, Keon Wook Kang

**Affiliations:** 1 Department of Nuclear Medicine, Seoul National University College of Medicine, Seoul, Republic of Korea; 2 Department of Biomedical Sciences, Seoul National University Graduate School, Seoul, Republic of Korea; 3 Tumor Biology Program, Seoul National University College of Medicine, Seoul, Republic of Korea; 4 Cancer Research Institute, Seoul National University College of Medicine, Seoul, Republic of Korea; 5 Biomedical Research Institute, Seoul National University Hospital, Seoul, Republic of Korea; 6 Department of Molecular Medicine and Biopharmaceutical Sciences, Graduated School of Convergence Science and Technology, Seoul National University, Seoul, Republic of Korea; 7 Tumor Microenvironment Global Core Research Center, Seoul National University, Seoul, Republic of Korea; 8 Institute of Radiation Medicine, Medical Research Center, Seoul National University Hospital, Seoul, Republic of Korea; 9 Cancer Imaging Center, Seoul National University Hospital, Seoul, Republic of Korea; 10 Department of Neurosurgery, Seoul National University Hospital, Seoul, Republic of Korea; 11 Ischemic/Hypoxic Disease Institute, Seoul National University College of Medicine, Seoul, Republic of Korea; 12 Department of Nuclear Medicine, National Cancer Center, Goyang, Republic of Korea

**Keywords:** human serum albumin, SPARC, cancer imaging, tumor targeting, glioma

## Abstract

Human serum albumin (HSA) is the most abundant plasma protein. The main reason for using HSA as a versatile tool for drug delivery is based on its ability to accumulate in tumors. However, the mechanism of albumin accumulation in tumors is not yet clear. Many researchers using HSA as a drug-carrier have focused on the passive tumor targeting by enhanced permeability and retention (EPR) effect, while other investigators proposed that albumin binding proteins mediate albumin accumulation in tumors. We investigated whether HSA accumulation in tumors is mediated by the EPR effect or by secreted protein acidic and rich in cysteine (SPARC), which is known to be an albumin-binding protein.

**Methods:** To investigate the role of SPARC on HSA accumulation in tumors, we compared HSA uptake in U87MG glioblastoma cells with different SPARC expression. U87MG cells generally express high levels of SPARC and were, therefore, used as SPARC-rich cells. SPARC-less U87MG (U87MG-shSPARC) cells were established by viral-shSPARC transduction. We detected cellular uptake of fluorescence-labeled HSA by confocal microscopy in U87MG and U87MG-shSPARC cells. To demonstrate the mechanism of HSA accumulation in tumors, we injected FNR648-labeled HSA and FITC-labeled dextran in U87MG and U87MG-shSPARC tumor-bearing mice and observed their micro-distribution in tumor tissues.

**Results:** HSA was internalized in cells by binding with SPARC *in vitro*. HSA accumulation in U87MG glioma was associated with SPARC expression *in vivo*. FITC-dextran was distributed in U87MG tumors in the vicinity of blood vessels. The distribution of HSA, on the other hand, was observed in the regions remote from blood vessels of U87MG tumor tissues but not in U87MG-shSPARC tumor tissues.

**Conclusion:** Our results demonstrate that the tumor-distribution of HSA is affected not only by the EPR-effect but also by SPARC expression. SPARC enhances HSA accumulation in U87MG glioma and mediates active targeting of HSA in tumors.

## Introduction

Human serum albumin (HSA) is the most abundant plasma protein in the human body accounting for 60% total plasma proteins. The advantages of albumin as a nanocarrier for clinical applications are water-solubility, biocompatibility, long half-life in plasma (about 20 days) and low toxicity 1-3. The main reason for using HSA as a versatile tool for drug delivery is based on its ability to accumulate in tumors by the passive targeting mechanism of enhanced permeability and retention (EPR) effect 4, 5. A fundamental feature of the EPR effect is the hyperpermeable tumor vasculature, which increases the permeability of particles 20-200 nm in size 6, 7. HSA can extravasate and accumulate in the interstitial space of tumors and can be trapped there due to the lack of lymphatic vessels in tumors 8-10. Many studies have focused on the accumulation of HSA as a drug carrier or tumor-targeting agent by EPR 11-14. Abraxane^®^ (nab-paclitaxel) is the first approved product based on HSA and the first nanotechnology-based chemotherapeutic agent 15. In another example, nab-paclitaxel has been shown to have improved antitumor activity and better tolerability compared to Taxol in phase III clinical trial 16. This albumin-based nanomedicine was approved by the US Food and Drug Administration (FDA) for the treatment of breast cancer in 2005 17, non-small cell lung cancer in 2012 18, and pancreatic cancer in 2013 19, 20. The success of nab-paclitaxel has shown the potential of albumin as a drug carrier for tumor imaging and therapy.

However, the mechanism of albumin accumulation in tumors is not well-defined. It is not clear whether albumin infiltrates into the tumor by the EPR effect or, albumin-binding proteins and receptors mediate its accumulation in tumors 14. It has been shown that albumin binds to the 60 kDa glycoprotein (gp60, gene name is *albondin*) receptor on the vascular endothelial cell surface and is transported to the tumor interstitium by transcytosis 21, 22. Alternatively, the secreted protein acidic and rich in cysteine (SPARC) was postulated to sequester albumin in tumor stroma and is partly associated with the tumor-specific uptake of albumin. SPARC is a member of a family of matricellular proteins that modulate cell-matrix interactions and important cellular functions such as proliferation, survival, and cell migration 23, 24. SPARC is also known as an albumin-binding protein and is highly expressed in various cancers 25, 26. Some studies have shown the role of SPARC in the uptake of albumin in tumors 27, 28; however, this concept is still controversial 29. In this study, we investigated the relationship between SPARC and HSA, and SPARC-mediated active targeting of HSA in U87MG glioma.

## Results

### Comparison of SPARC expression and HSA accumulation in various cancer cells

We examined SPARC mRNA and protein expression in various cancer cell lines. Because glioma has been reported to be high SPARC-expressing cancer in patients 27, we tested several glioma as well as other cancer cell lines. Glioma cell lines (U87MG, U373, and U251) showed high SPARC expression (Figure S1A). Since SPARC is known to be a secreted protein, we measured the level of SPARC secreted into the serum-free medium and found it to be highly expressed in and secreted by glioma cells (Figure S1B). We chose U87MG cells to investigate the role of SPARC in HSA uptake. To visualize HSA uptake by glioma cells, we first prepared fluorescence (FNR648)-labeled HSA and treated U87MG cells with FNR648-HSA. As shown in Figure S1C and D, high accumulation of FNR648-HSA in SPARC-expressing cells, especially in glioma cells, was observed.

### The role of SPARC in HSA cellular uptake

**Figure 1:**
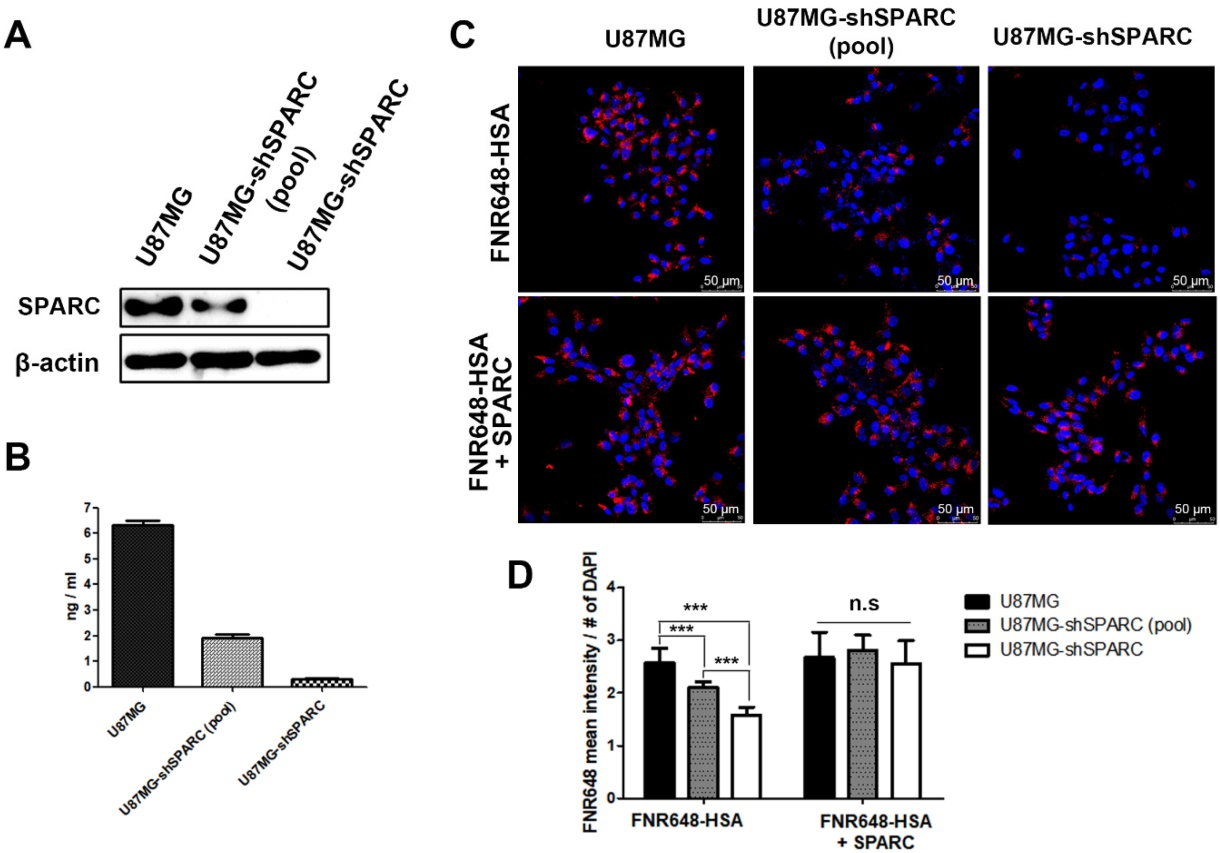
SPARC protein expression and FNR648-HSA uptake in U87MG cells expressing different levels of SPARC. SPARC expression in (A) Cell lysates and (B) conditioned serum-free cell media. (C) Representative cell images after FNR648-HSA treatment. To demonstrate the effect of SPARC on intracellular uptake of HSA, SPARC was treated with FNR648-HSA. Scale bar, 50 μm. (D) Quantification of FNR648-HSA uptake in each cell type from confocal images. ***: P < 0.001.

To further verify the relationship between SPARC and HSA uptake in U87MG cells, SPARC shRNA lentiviral transduction was used to generate SPARC down-regulated U87MG cells. The lowest SPARC-expressing U87MG cell clone (1C3 clone) was selected and labeled as U87MG-shSPARC (Figure S2). Using U87MG, U87MG-shSPARC (pool) and U87MG-shSPARC cells, we examined whether HSA uptake in cells could be affected by SPARC. SPARC protein levels in cells (Figure 1A) and serum-free media (Figure 1B) were highest in U87MG cells and lowest in U87MG-shSPARC cells. For cell uptake studies of HSA, U87MG and U87MG-shSPARC cells (Figure S2) were treated with FNR648-HSA for 2 h, and HSA uptake patterns were observed. As can be seen in Figure 1C, U87MG took up more FNR648-HSA than U87MG-shSPARC cells.

To confirm the effect of SPARC on HSA uptake, three cell lines were co-treated with human SPARC and FNR648-HSA. SPARC co-treatment specifically increased FNR648-HSA uptake in U87MG-shSPARC cells (Figure 1C, FNR648-HSA + SPARC). It was evident from quantification data that FNR648-HSA uptake was proportional to the SPARC expression level in at least five images from three different samples from each group (Figure 1D). After exogenous SPARC co-treatment, FNR648-HSA uptake was enhanced in U87MG-shSPARC cells (pool) and U87MG-shSPARC cells but was unchanged in U87MG cells (Figure 1D, n.s). These results showed that SPARC facilitates the uptake of HSA in cells.

### Co-localization of SPARC and HSA in U87MG cells

**Figure 2:**
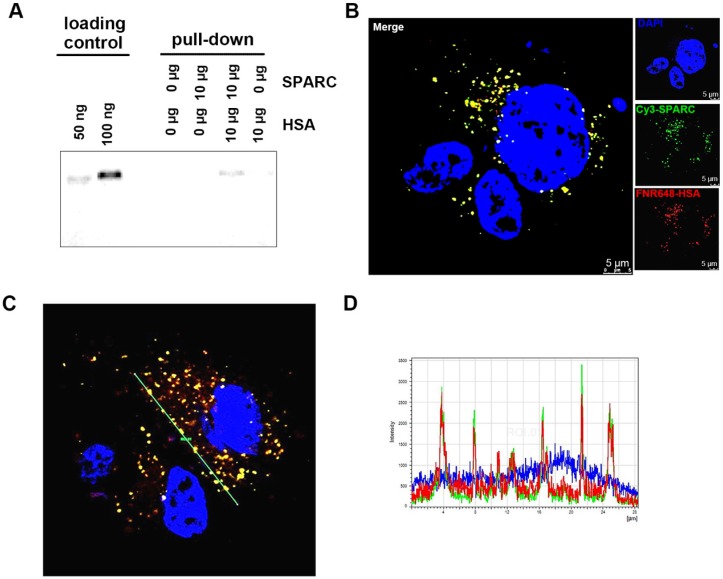
Confirmation of binding of HSA and SPARC at the protein and cellular levels. (A) Fluorescence image of immunoprecipitated samples. SPARC antibody was used and SPARC-bound albumin was detected by FNR648. (B-C) Fluorescence imaging of Cy3-SPARC- and FNR648-HSA-treated cells. To visualize the co-localization of SPARC and HSA in cells, FRET methodology was used in U87MG cells (B-C); Cy3-SPARC (λ excitation: 514 nm; λ emission: 540-600 nm) and FNR648-HSA (λ emission: 640-750 nm). (B) Representative FRET images. (C) Representative line ROI analysis of the signal. The green line represents the line ROI in the confocal images. (D) Signal intensity vs. location graph from (C). Line ROI signal graph shows co-localization of Cy3-SPARC and FNR648-HSA in cells. Scale bar, in (B) 5 μm.

That exogenous SPARC treatment could increase HSA uptake in glioma cells (Figure 1) prompted us to examine if the binding of SPARC to HSA mediated its uptake by tumor cells. We used a pull-down assay to test the affinity between HSA and SPARC. We immunoprecipitated a mixture of SPARC and FNR648-HSA after incubation for 2 h to allow binding between the two proteins. Following the pull-down, samples were immunoprecipitated using anti-SPARC antibody, loaded onto gels, and fluorescence signals were measured (Figure 2A). Fluorescent HSA was observed only in SPARC and HSA incubation samples at a location identical with the control FNR648-HSA confirming that HSA could bind to SPARC.

To ascertain that HSA binds to SPARC and enters into the cells, we used fluorescence resonance energy transfer (FRET) to observe mutual binding of HSA and SPARC 30, 31 and used Cy3 as a donor and FNR648 (Excitation λ_max_: 648nm) as an acceptor. Cy3-SPARC and FNR648-HSA were detected by using their respective FRET wavelengths (Figure 2B, Cy3-SPARC as green color and FNR648-HSA as red color). The merged image showed a strong yellow signal at the location of SPARC and HSA in cells (Figure 2B, Merge). Also, a line ROI analysis of signals in the FRET image showed that the location of Cy3-SPARC (intensity peaks) matched with that of FNR648-HSA (Figure 2C). The co-localized Cy3-SPARC and FNR648-HSA were not in the nucleus but in the cytosol (Figure S4). The overlapping images of Cy3-SPARC and FNR648-HSA (less than 10 nm distance) suggested that HSA moved into the cytosol by binding to SPARC.

### *In vivo* imaging of FNR648-HSA in U87MG glioma xenograft tumor model

**Figure 3:**
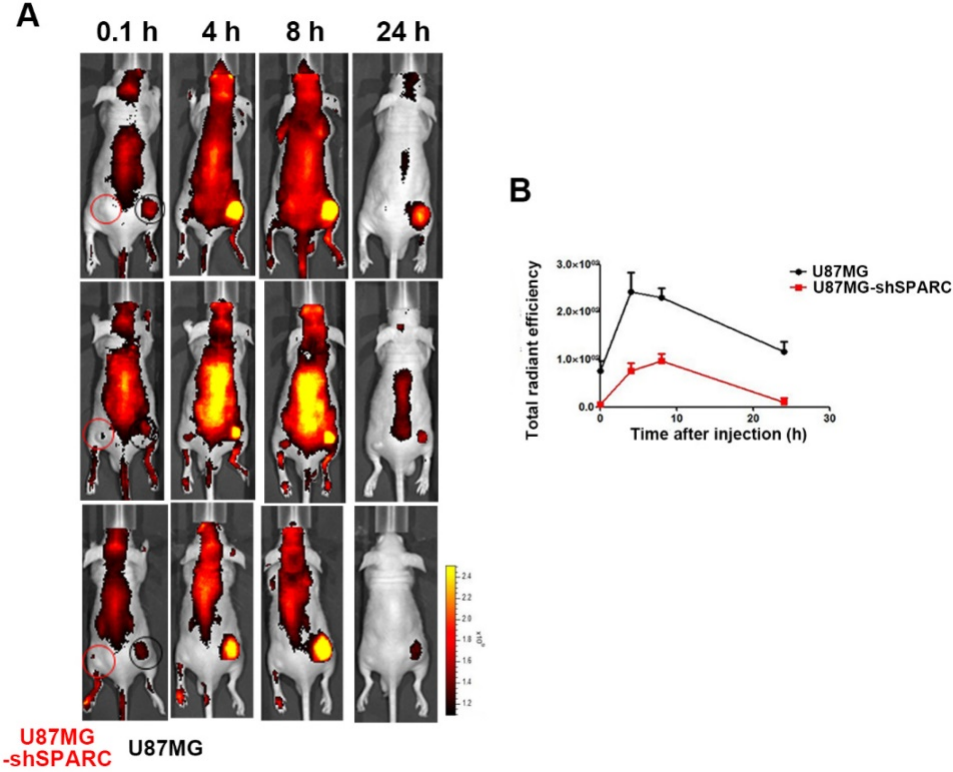
Distribution of FNR648-HSA in tumor-bearing mice. FNR648-HSA was injected into U87MG (Right-thigh) and U87MG-shSPARC (Left-thigh) xenografted tumor-bearing mice. (A) FNR648-HSA images in tumor-bearing mice. The distribution of FNR648-HSA in mice was acquired at various time points after injection. (B) To monitor FNR648-HSA accumulation in tumors, fluorescence signals from tumors were acquired through ROI analyses (n = 3). The image signal unit was total radiant efficacy in [photons/sec]/[μW/cm^2^].

Since we observed that HSA binding to SPARC could affect its accumulation in cells *in vitro*, we verified the effect of SPARC on HSA accumulation in an *in vivo* tumor model. We observed the distribution of FNR648-HSA in glioma tumors in mice. FNR648-HSA was injected through the tail vein in U87MG and U87MG-shSPARC xenograft tumor-bearing mice. Fluorescence signal images were obtained at 0.1, 4, 8, and 24 h after injection which showed stronger fluorescence signal in U87MG tumors than U87MG-shSPARC tumors (Figure 3A). HSA accumulation was analyzed at each time point by the fluorescence signals in tumors using ROI analyses. The results showed higher fluorescence signals in U87MG tumors than U87MG-shRNA tumors at all time points. The highest fluorescence signal in U87MG tumors was observed at 4 h after injection (Figure 3B). These results indicated that SPARC expression affects the HSA accumulation in tumors *in vivo*.

### Tumor accumulation of FNR648-HSA and FITC-dextran in mice

**Figure 4:**
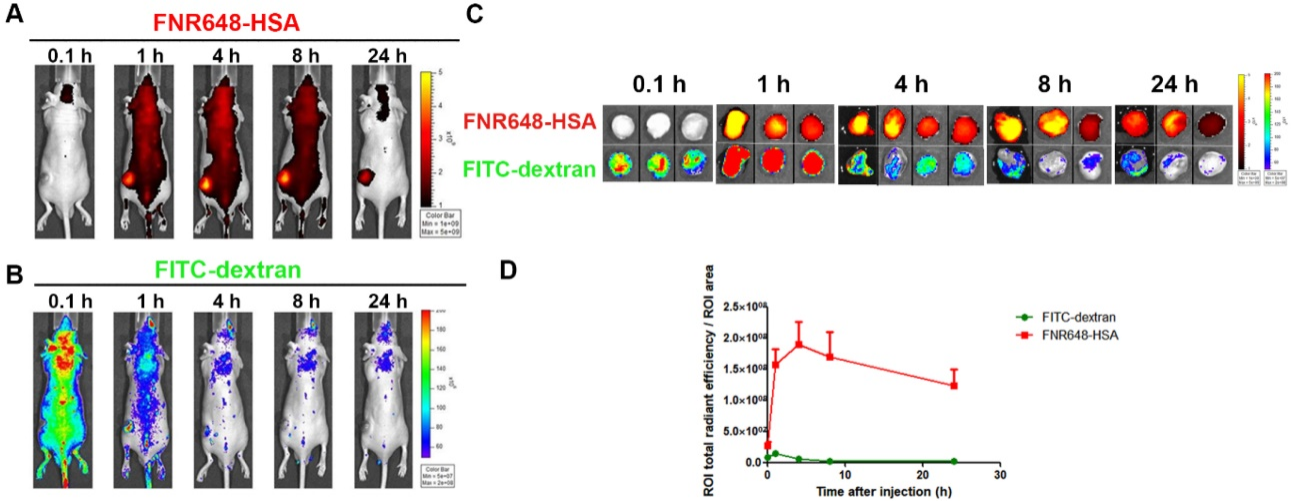
Distribution of FNR648-HSA and FITC-dextran in U87MG tumor-bearing mice. Time-course images of mice after intravenous injection of FNR648-HSA and FITC-dextran. (A) FNR648-HSA images and (B) FITC-dextran images. (C) *Ex vivo* images of tumors acquired at indicated time-points after FNR648-HSA and FITC-dextran injections. Tumor images were acquired at each time-point after sacrificing the mice (n = 3 for 0.1, 1, 8, 24 h after injection and n = 4 for 4 h). (D) Fluorescence signal analysis using *ex vivo* tumors. Mice whole body images for tumor *ex-vivo* signal analysis are shown in Figure S6.

To investigate the mechanism of FNR648-HSA accumulation in tumors, we co-injected FNR648-HSA and FITC-dextran in U87MG tumor-bearing mice. Since the EPR effect is dependent on the particle size and molecular weight, FITC-dextran with 40 and 70 kDa-dextran showed the highest tumor accumulation by the EPR-effect 7. Because of its similar size to HSA (HSA: 63 kDa), 70 kDa FITC-dextran was selected for *in vivo* comparative tumor accumulation. Dextran-signals were decreased with time (Figure 4B), but FNR648-HSA was gradually increased up to 4 h after injection decreasing thereafter (Figure 4A). We also excised mouse tumor tissues at each time point (0.1, 1, 4, 8, 24 h after injection), and *ex-vivo* tumor images were acquired (Figure 4C) to analyze FNR648-HSA and FITC-dextran signals in tumors over time (Figure 4D). FITC-dextran showed the highest accumulation in tumors at 1 h after injection, while the fluorescence intensity of FNR648-HSA was the highest at 4 h after injection. Fluorescence signals in tumor tissues were observed using confocal microscopy and this signal pattern (versus time) was consistent with ROI analysis (Figure S5).

### Micro-distribution of FNR648-HSA and FITC-dextran in tumor tissues

**Figure 5:**
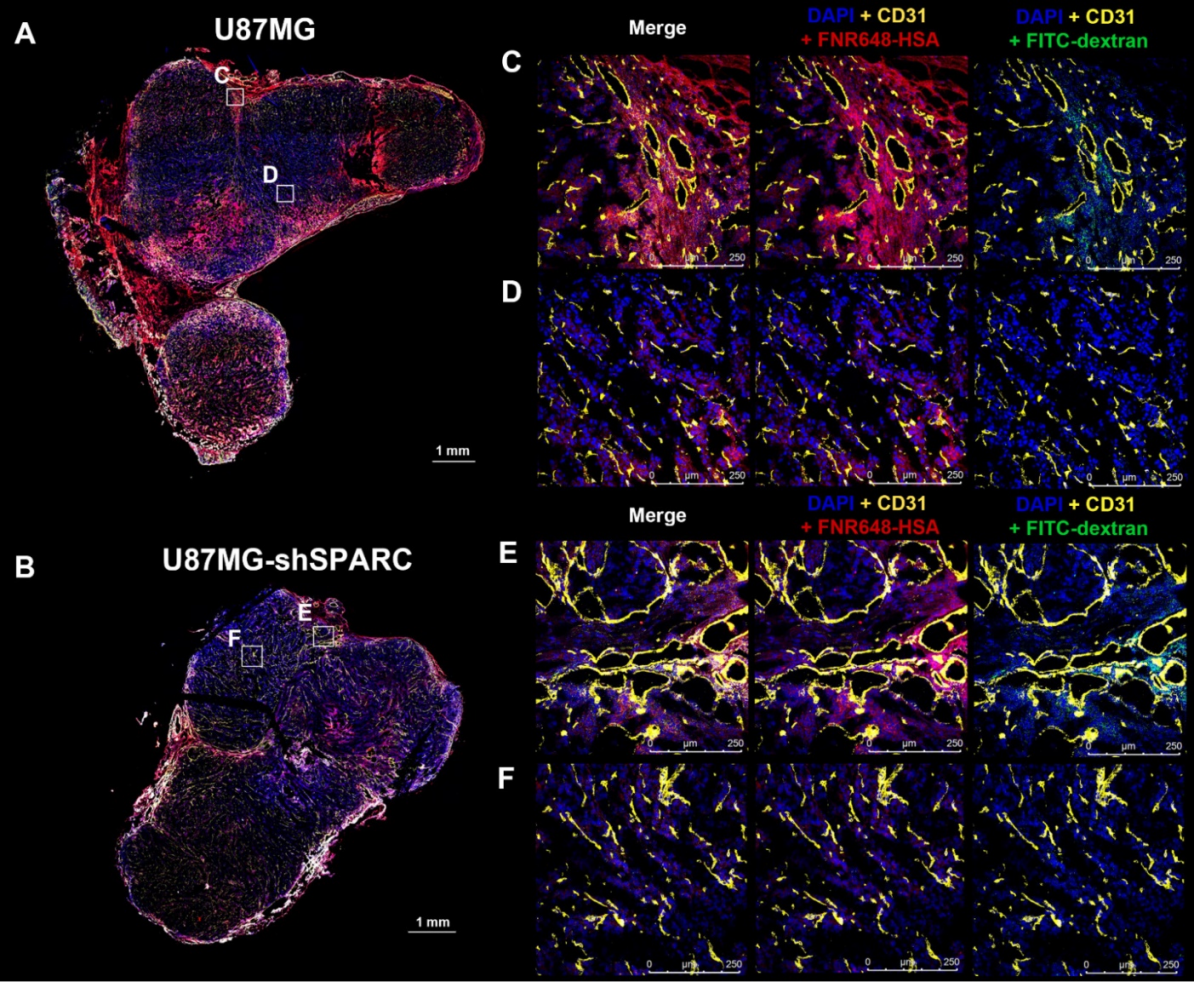
Micro-distribution of FNR648-HSA and FITC-dextran in tumor tissues. Immuno-fluorescence staining and confocal images were obtained using frozen-tumor section. Total tumor immunofluorescence image of (A) U87MGB and (B) U87MG-shSPARC. The blood vessels and cell nuclei were stained with anti-CD31 antibody and DAPI, respectively. All images are merged images. The white square represents the enlarged region for each image (C, D, E, F for each image). Separated fluorescence signal images (DAPI, CD31, HSA, and FITC-dextran) from the white squares are shown in Figure S7. Each image is labeled with its scale bar, 1 mm or 250 μm.

We monitored the differences in micro-distribution of FNR648-HSA and FITC-dextran in tumor tissues. Tumor tissues were acquired from mice at 4 h after co-injection of FNR648-HSA and FITC-dextran and tumor sections were stained with CD31 for blood vessels. FITC-dextran was observed in the vicinity of blood vessels (CD31) in U87MG and U87MG-shSPARC tumors (Figure 5C-F; DAPI + CD31 + FITC-dextran and Figure S7A-D; FITC-dextran as green signals). In the U87MG tumor, FNR648-HSA was observed not only in the blood vessel regions, (Figure 4C; DAPI + CD31 + FNR648-HSA, and Figure S7A; FNR648-HSA as red signals) but also in the regions remote from the blood vessels (Figure 4D; DAPI + CD31 + FNR648-HSA and Figure S7B; FNR648-HSA as red signals). On the contrary, in the U87MG-shSPARC tumor, FNR648-HSA was located in the vicinity of blood vessels (Figure 4D; DAPI + CD31 + FNR648-HSA, and Figure S7B; FNR648-HSA as red signals) and not in remote regions from the blood vessels (Figure 4F; DAPI + CD31 + FNR648-HSA, and Figure S7D; FNR648-HSA as red signals).

To investigate the correlation between FNR648-HSA distribution and SPARC, we examined SPARC expression in tumors stained with anti-human SPARC antibody (Figure S8). U87MG tumors showed higher expression of SPARC in tumor tissues than U87MG-shSPARC tumors (Figure S8A, B). In U87MG tumors, FNR648-HSA accumulation in the regions remote from the vessels correlated with SPARC expression (Figure S8D).

## Discussion

It is still unclear whether the mechanism associated with HSA uptake in tumors is merely due to the passive targeting by the EPR effect, or active targeting is also involved. Many studies using HSA as a tumor imaging agent or a drug-delivery system have just focused on HSA accumulation in tumors and have not been able to explain its mechanism.

In this study, we first showed HSA accumulation in U87MG glioma by SPARC-mediated active targeting of HSA. We visualized HSA uptake in various cancer cells, especially glioma cells expressing more SPARC showed higher HSA uptake. Based on these observations, we chose U87MG glioma to further elucidate the relationship between SPARC and HSA. We also showed that the high uptake of HSA in U87MG was dependent on SPARC expression. This observation was verified by exogenous SPARC treatment of U87MG-shSPARC cells.

To verify that the HSA uptake was dependent on its binding to SPARC, we employed FRET analysis. The FRET images clearly showed the co-localization of HSA and SPARC in cells. Thus, from the* in vitro* experiments of cellular uptake of HSA and the FRET analysis, we provided strong evidence that SPARC is closely associated with HSA.

Previous studies have shown a correlation between SPARC and HSA at the protein expression level in cancer cells and possible implications for the therapeutic effect but did not show a direct relationship of SPARC in HSA accumulation in tumors 32. Another study showed the binding affinity between bovine serum albumin (BSA) and SPARC, and its internalization using a non-cancer cell line and suggested that HSA may have a similar affinity to SPARC 33. Therefore, it was necessary to identify HSA and SPARC interactions in cancer cells. In this study, we showed binding between HSA and SPARC using immunoprecipitation. We also confirmed the direct relationship between SPARC and HSA in cells using the FRET technique which demonstrated the *in vitro* binding between SPARC and HSA. Most importantly, we observed SPARC-mediated HSA accumulation in tumor tissues. To identify the active targeting of HSA in tumor accumulation, we co-injected FNR648-HSA with FITC-dextran (70 kDa), which represents the EPR-mediated nanoparticle accumulation in tumors and compared the distribution patterns of HSA and FITC-dextran. In U87MG tumor tissue expressing SPARC, the HSA distribution was similar to but also apparently different from that of FITC-dextran. FNR648-HSA was observed not only in the blood vessel regions but also in the regions distant from vessels and its distribution pattern in the regions away from vessels correlated with SPARC expression in U87MG tumor. However, in U87MG-shSPARC tumor tissues, HSA was detected only in the vicinity of blood vessels in a pattern that was similar to FITC-dextran distribution in tumors. It was evident that SPARC enhanced the HSA targeting effect on U87MG glioma. These results indicated that HSA could accumulate in tumors not only by the EPR effect but also by active targeting through SPARC expression.

We also evaluated HSA accumulation in U87MG or U87MG-shSPARC tumor xenograft models. U87MG, expressing a higher level of the SPARC protein, showed greater accumulation of HSA in tumors than U87MG-shSPARC. U87MG-shSPARC tumors also showed somewhat increased uptake at 4 h after injection which can be explained by the following reasons. First, HSA could accumulate in U87MG-shSPARC tumors by the EPR effect. Second, from *in vitro* experiments, it was clear that shSPARC transduction lowered SPARC protein expression in U87MG-shSPARC cells (1C3) but did not completely eliminate it (Figure S2A). The residual SPARC protein expression in the U87MG-shSPARC xenograft tumors may thus affect the HSA uptake *in vivo*. When an anti-human SPARC antibody was used, SPARC expression was low in U87MG-shSPARC tumor tissues (Figure S8B and E). We also observed that HSA accumulation in tumors was decreased 4 h after injection (Figure 3). This could be explained by the observation that growing tumor tissues use albumin as a source of amino acids and energy 34.

In this study, we used subcutaneous glioma xenograft model for tumor accumulation of HSA. It is well known that subcutaneous tumor model is different to naturally occurring tumor. For example, xenograft tumor can be developed within a short period of time and has rich vasculature 35. As a result, subcutaneous tumor model shows more EPR effect in tumors. To verify SPARC-mediated HSA accumulation in tumors is a common phenomenon, it is necessary to use other naturally occurring cancer models, such as genetically engineered, spontaneous carcinogenesis model.

SPARC was first identified as osteonectin, a bone-specific phosphoprotein that binds to collagen fibrils. Previous studies on SPARC have shown its extracellular matrix-related function 36-38. The role of SPARC in tumors is very different among tumor types 39. Especially in gliomas, SPARC is highly expressed 40 and inhibits tumor growth, but induces dissociation and increased migration 41, 42. The aspect of SPARC as tumor growth is seem to condition dependent, because SPARC also increase cell survival under serum-withdrawal condition 43 or chemotherapy treatment 44. The main characteristics of glioma are intratumor heterogeneity and highly invasive nature 45. It is known that glioma progression and differentiation is associated with tumor heterogeneity and tumor microenvironment, but the detailed mechanism is poorly understood. Because SPARC is secreted form of protein, it can affect to tumor microenvironment 46. One research showed that the loss of SPARC in astrocytes which is null for p53 results in reduced tumor formation and increased tumor immunogenicity in brain tumor model 47. SPARC also expressed from tumor microenvironmental components, such as endothelial cells, and this non-cancer cell originated SPARC also affect to glioma. A recent study has shown that high-grade glioma spreads to the lateral ventricle subventricular zone through a chemoattractant multimeric protein complex of which SPARC is a component 48. Research about the interaction of tumor and microenvironment by SPARC is not well understood, so this topic has attracted much attention and is especially important in glioma.

In this study, we have shown that SPARC mediates HSA accumulation in U87MG glioma cells that express high levels of SPARC. It is known that there are two different pathways of SPARC mediated cellular uptake. One is extracellular SPARC mediated pathway, which might rely on the rapid exchanged of lipidic components 27, and the other is cancer cell membrane SPARC mediated pathway, which is receptor-mediated endocytosis 49. Further studies are needed to distinct two pathways of SPARC mediated uptake of HSA.

Other molecular mechanisms of non-SPARC-mediated HSA accumulation in tumors cannot be ruled out. One study showed that caveolae-mediated endocytosis is critical for HSA and nab-paclitaxel uptake in tumors 50. Also, gp60 (*albondin*) is known to be involved in caveolin-1 mediated endocytosis in endothelial cells 14. Because of the protein homology between gp60 (*albondin*) and SPARC, it is worthwhile to investigate the possible relation between SPARC and caveolin. Another study reported that SPARC was efficiently endocytosed by stabilin-1-mediated endocytosis in activated macrophages 51. It is evident that, in the future, other potential underlying molecular mechanisms of SPARC-mediated HSA accumulation in tumor cells should be investigated for its effective application as a nanocarrier for targeting tumors.

Other receptors affect HSA uptake in cells including the neonatal Fc receptor (FcRn) 52, which is known to bind albumin in a pH-dependent manner and affects the half-life of serum albumin *in vivo* 53. Since FcRn function may affect the albumin accumulation in tumors, we also examined its expression at the cellular level (Figure S10) and found similar levels of expression in U87MG and U87MG-shSPARC cells.

Currently, the first-line standard therapy of glioma is maximally safe surgical resection. Since gliomas are invasive and often present in important areas of the brain, such as speech control, motor function, and the senses, extensive and complete surgical resection of glioma is difficult 54. Our study has shown the theranostic potential of HSA for targeting glioma both for imaging and as a therapeutic agent. HSA-mediated anticancer-drug delivery to glioma after surgical resection represents a potentially promising strategy for eliminating the remnant tumor.

## Conclusion

HSA accumulation in U87MG glioma correlates with SPARC expression *in vitro* and *in vivo*. Tumor accumulation and micro-distribution of HSA in glioma were enhanced by SPARC. These results suggest that SPARC mediates active targeting of HSA in U87MG gliomas and, therefore, HSA-mediated anti-cancer drug delivery for highly malignant gliomas and other SPARC-expressing cancers has great potential in the clinic.

## Materials and Methods

### Cells

Human prostate cancer cell line PC3, human lung cancer cell line A549, and human breast cancer cell line MDA-MB-231 were purchased from the Korean Cell Line Bank (Seoul, Korea). Human glioma cells (U251, U373, and U87MG) were purchased from ATCC (American Type Culture Collection, Manassas, VA). U251 and MDA-MB-231 were maintained in Dulbecco's modified Eagle medium (DMEM, WELGENE, Gyeongsan, Korea). PC3 and A549 were maintained in Roswell Park Memorial Institute 1640 medium (RPMI1640, WELGENE). U87MG and U373 were grown in Minimum Essential Medium (MEM, Gibco, Grand Island, NY). Each medium (DMEM, MEM, and RPMI) was supplemented with 10% (v/v) fetal bovine serum (FBS, Gibco) and 1% antibiotics containing penicillin/streptomycin (Invitrogen, Grand Island, NY). All cells were maintained at 37 °C in a humidified atmosphere with 5% CO_2_.

### Establishment of low SPARC-expressing U87MG cells

U87MG cells were stably transduced with SPARC shRNA (human) lentiviral particles (sc-37166-V, Santa Cruz Biotechnology (SCB), Dallas, TX) according to the manufacturer's protocol.

### RT-PCR

Total RNA was isolated from cells using TRIzol reagent (Invitrogen) according to the manufacturer's protocol. For cDNA synthesis, amfiRivert Platinum cDNA synthesis Master Mix (GenDEPOT, Barker, TX) was used with 2 μg of mRNA, as per the manufacturer's instructions. From the synthesized cDNA, mRNA expression levels of SPARC and β-actin were detected. The primer sequences for the SPARC gene were 5′-GGT ATC TGT GGG AGC TAA TC-3′ (forward) and 5′-TCT CAG TCA GAA GGT TGT TG-3′ (reverse). As an endogenous control, the β-actin gene with the primer sequences 5′-ACC AGG GCT GCT TTT AAC TCT-3′ (forward) and 5′-GAG TCC TTC CAC GAT ACC AAA-3′ (reverse) was used. For amplification of SPARC and β-actin, PCR reactions were performed using i-MAX Ⅱ DNA polymerase (iNtRON Biotechnology, Daejeon, Korea) as follows: 94 °C/5 min, followed by 30 cycles of 94 °C/30 s, 53 °C/30 s, and 72 °C/min; elongation step was at 72 °C for 10 min. PCR products were analyzed by gel electrophoresis in 1.2% agarose gels and visualized using loading STAR (Dyne Bio Inc., Seoul, Korea) staining. The product sizes of SPARC and β-actin were 452 bp and 225 bp, respectively.

### Western blotting

Total cell protein was extracted with radioimmunoprecipitation assay (RIPA) buffer (Sigma-Aldrich, St. Louis, MO) containing a protease inhibitor cocktail (Roche, Basel, Schweiz). After centrifugation at 13000 rpm for 20 min, supernatants were collected, and protein concentration was measured with a bicinchoninic acid (BCA) assay kit (Pierce, Thermo Fisher Scientific, Waltham, MA). Proteins (20 μg) were separated by 10% SDS-PAGE and transferred to polyvinylidene difluoride membranes (Millipore, Billerica, MA). The protein-loaded membranes were blocked with 5% skim milk in Tris-buffered saline containing Tween-20 (TBST) for 1 h at room temperature, followed by incubation with the primary antibodies against SPARC (5420S, 1: 1000 dilution, Cell Signaling Technology, Danvers, MA), FcRn (sc-271745, 1: 200 dilution, SCB), and β-actin (A5441, 1: 10000 dilution, Sigma-Aldrich) at 4 °C overnight. Horseradish peroxidase-conjugated anti-rabbit and anti-mouse antibodies (1: 4000 dilution, CST) were subsequently used as secondary antibodies. Proteins were detected with an enhanced chemiluminescence kit (Pierce) using the LAS4000 imaging system (Fujifilm, Tokyo, Japan).

### ELISA

We seeded 1.5 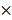 10^5^ cells/well in 6-well plates for 24 h. For quantifying SPARC level, the culture medium was replaced with serum-free medium. Subsequently, the culture medium was collected after 24 h for measuring the secreted form of SPARC. Haman SPARC Quantikine ELISA kit (DSP00, R&D system, Minneapolis, MN, USA) was used for detecting the secreted form of SPARC measurement.

### Preparation of fluorescence dye-labeled HSA and SPARC

We conjugated HSA with FNR648 fluorescence dye following the procedure described in our previous publication 55.

For fluorescence dye labeling of SPARC, recombinant human SPARC (cell-derived, 941-SP, R&D system) was modified using ADIBO-NHS for fluorescence dye labeling. The synthetic procedure for clickable modified SPARC was performed in two steps. First, 50 μg of SPARC dissolved in 0. 5 mL PBS was mixed with 1 μL of ADIBO-NHS dissolved in DMSO (10 mM). The reaction time was 30 min at RT and then 1 h at 4℃. The ADIBO-conjugated SPARC (ADIBO-SPARC) was washed 3 times with PBS by centrifuging each time at 13000 rpm for 10 min, at 4 °C. Subsequently, Cy3-azide (Sigma-Aldrich, St. Louis, MO, USA) was mixed with ADIBO-SPARC using click reaction for 1 h. Fluorescence-labeled SPARC was washed with PBS 3 times and centrifuged at 13000 rpm for 10 min at 4 ℃. The final volume of 0.1 mL was used to determine the concentration using NanoDrop^TM^ (Thermo Fisher Scientific, Waltham, MA).

### *In-vitro* FNR648-HSA cellular uptake

Cells were seeded on cover-glass (Paul Marienfeld GmbH & Co. KG, Lauda-Königshofen, Germany) in 12-well plates (Nalge NUNC International, Naperville, IL) at a density of 1 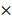 10^5^ cells/well. Subsequently, the cells were treated with FNR648-HSA (2 nmole/well) in each cell-culture medium and incubated at 37 °C for 0. 5 h or 2 h. Recombinant human SPARC (cell-derived, 941-SP, R&D system, Minneapolis, MN) was purchased for observing the SPARC effect on HSA uptake. For the exogenous human SPARC treatment (5 μg/ml) group, human SPARC and FNR648-HSA were co-incubated with cells at 37 °C for 0. 5 and 2 h. After each incubation time, cells were removed from FNR648-HSA-added medium and washed with PBS twice followed by a fixation procedure using paraformaldehyde (PFA, SCB) for 10 min. Cover glasses were removed from 12-well plates and mounted with ProLong Gold reagent (Invitrogen) on glass slides (Paul Marienfeld GmbH & Co. KG). Five randomized images were acquired from all tests as quantification of FNR648-HSA uptake by the cells. Three independent tests were performed for the cell uptake experiment. The average signal intensity of FNR648-HSA in the image was measured using the LAS X program and the numbers of DAPI in the image were counted. The average signal intensity of FNR648-HSA was divided by the number of DAPI, which represented the number of viable cells. We considered this ratio as FNR648-HSA uptake in cells.

### Flow cytometry (FACS)

Cell uptake procedure was same as *in vitro* FNR648-HSA cellular uptake. After incubation of FNR648-HSA with cells, cells were detached from plate and move into test tube for fluorescence signal analysis. FACS canto Ⅱ (BD Biosciences, San Jose, CA) was used for flow cytometry signal acquirement from cells. Data was analyzed using FlowJo (BD bioscience).

### Immunoprecipitation (IP)

BSA and SPARC binding was determined using IP methods following the previously published procedure 33. For the pre-clearing step, we incubated 10 μg of HSA, 4.76 μg/mL of normal rabbit serum (X0902, Dako, Santa Clara, CA), and 20 μl of protein A/G PLUS-Agarose (sc-2003, SCB) in a test tube for 1 h at 4 °C. After centrifugation, SPARC and HSA were incubated for 2 h at 4 °C. Rabbit anti-SPARC antibody (5420S, CST, diluted 1: 50) was added and incubated for 2 h followed by incubation for an additional 2 h with protein A/G PLUS-Agarose. Complexes were centrifuged and loaded onto 8% SDS-PAGE gel to detect FNR648-HSA signal. Fluorescence signal in the gel was acquired using IVIS (PerkinElmer, Waltham, MA).

### Animal xenograft modeling and IVIS imaging

Animal studies were performed according to the National Research Council guidelines of the Seoul National University Hospital (IACUC No.; 15-0279). BALB/C nude mice (male, 6-week old) were used for in-vivo xenograft-tumor modeling. U87MG and U87MG-shSPARC cells were subcutaneously injected into both thighs in mice, at 5 × 10^6^ cells. Tumor size and mouse weight were regularly measured. Approximately 2 weeks after tumor inoculation in mice, *in-vivo* FNR648-HSA imaging experiments were conducted using IVIS Lumina Ⅱ (PerkinElmer, Waltham, MA). Mice were injected with FNR648-HSA (80 μg/200 μL PBS to each mouse) and fluorescence signals were recorded. For FNR648-HSA and 70-kDa FITC-dextran (Sigma-Aldrich) co-injection group, FITC-dextran was injected at 1 mg/40 μL per mouse. Gray-scale photographs of the mouse and corresponding pseudo-color images were superimposed with Living Image, version 2.12 (Xenogen, Alameda, CA) and IGOR, version 1.24 (WaveMetrics, Portland, OR) image analysis software.

### Immunofluorescence staining

Tumors were harvested from sacrificed mice 4 h after FNR648-HSA injection and embedded in OCT compound (Leica Biosystems, Richmond, IL). After 24 h freezing procedure in an ultra-cold freezer (-80 °C), the specimens were cut into 4-μm thickness using Leica CM3050 S cryostat (Leica, Wetzlar, Hesse, Germany). Frozen tumor slides were thawed at RT for 5 min, followed by washing the slides thrice with PBS, for 5 min each. This was followed by fixation using paraformaldehyde (SCB) for 15 min and rewashing the slides thrice with PBS, for 5 min each. The cells on the slides were permeabilized with 0.5% Triton X-100 for 15 min. After the permeabilization step, the samples were washed thrice with PBS and blocked subsequently with 10% normal serum and 1% BSA in TBS for 1 h at RT. Next, the cells were treated with anti-CD31 primary antibody (AF3628, R&D system, diluted 1: 100) or anti-human SPARC antibody (AF941, R&D system, diluted 1: 200) in 1% BSA, TBS, and incubated at 4 °C overnight. On the following day, the cells were incubated with secondary antibody, diluted 1: 400 in 1% BSA, TBS, for 2 h at room temperature, and finally stained with DAPI.

### Confocal microscopy imaging

In the *in-vitro* cell uptake assay or immunofluorescence staining, confocal microscopy imaging was used to analyze the fluorescence signal from the samples. Fluorescence images were taken by Leica TCS SP8 confocal laser scanning microscope (Leica); fluorescence signal was detected in the specific range of wavelengths (DAPI; 401-480 nm, FITC; 500-550 nm, TRITC; 580-600 nm, FNR648; 650-750 nm). For FRET imaging, the excitation wavelength was 514 nm and the emission wavelength 540-600 nm for Cy3-SPARC and 640-750 nm for FNR648-HSA.

### Statistical analysis

Data were analyzed as the mean ± standard deviation and using the Mann-Whitney U test. A P value of less than 0.05, 0.01 or 0.001 was considered statistically significant. Statistical analyses were performed using GraphPad Prism version 5.0 (GraphPad).

## Supplementary Material

Supplementary figures and tables.

## Abbreviations

SPARC: secreted protein acidic and rich in cysteine
BSA: bovine serum albumin
HSA: human serum albumin
FITC: fluorescein isothiocyanate
EPR: enhanced permeability and retention
gp60: 60 kDa glycoprotein
IP: immunoprecipitation
FRET: fluorescence resonance energy transfer
ROI: region of interest
DAPI: 4',6-diamidino-2-phenylindole
