# Supplementary material for: Secreted protein acidic and rich in cysteine mediates active targeting of human serum albumin in U87MG xenograft mouse models
Source: Theranostics. 2019 Oct 11;9(24):7447–57. doi: 10.7150/thno.34883 (PMC6831305; doi:10.7150/thno.34883)
Supplement: Supplementary file 1 — Supplementary figures and tables. [file thnov09p7447s1.pdf]

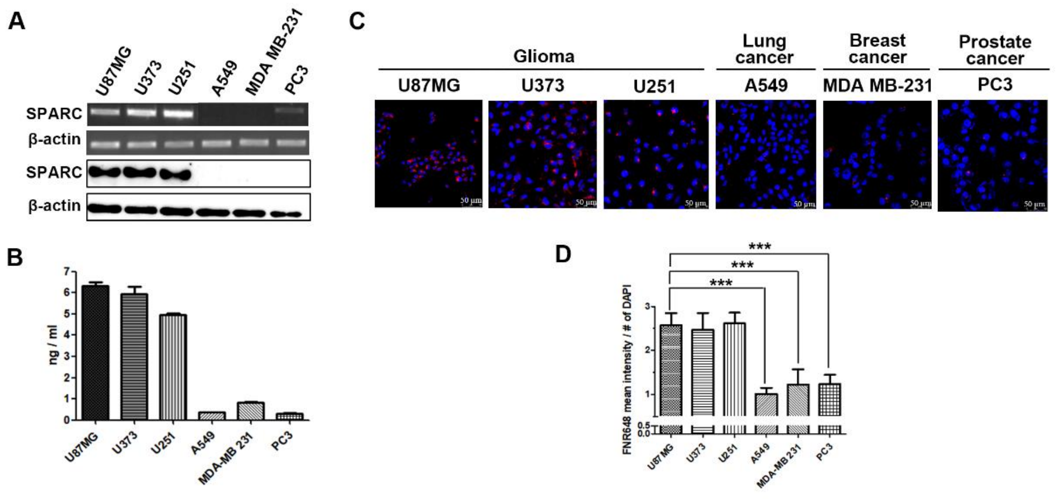

Figure S1. SPARC expression and HSA uptake in various cancer cells. (A) SPARC mRNA and protein expression levels. (B) Levels of secreted SPARC in serum-free media. (C) Fluorescence images of FNR648-HSA. FNR648-HSA is shown in red color. Nuclear staining was performed with DAPI. Scale bar, 50 μm. (D) Quantification of FNR648-HSA uptake in each cell line from confocal images. ***: P < 0.001.

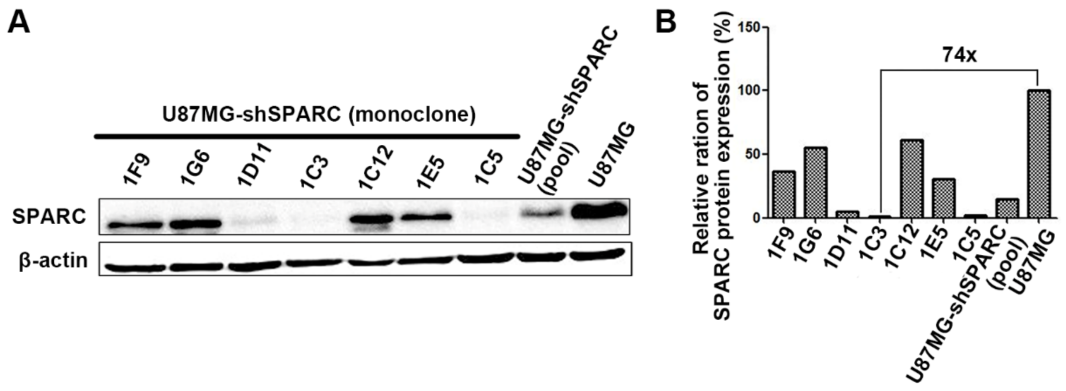

Figure S2. SPARC protein expression in SPARC shRNA-transduced cells. (A) SPARC protein expression level in cells of individual clones from U87MG-shSPARC pool. (B) Relative SPARC expression in individual clones selected from U87MG-shSPARC cells. The

lowest SPARC-expressing cells (1C3) showed 74-fold lower protein expression level than U87MG cells.

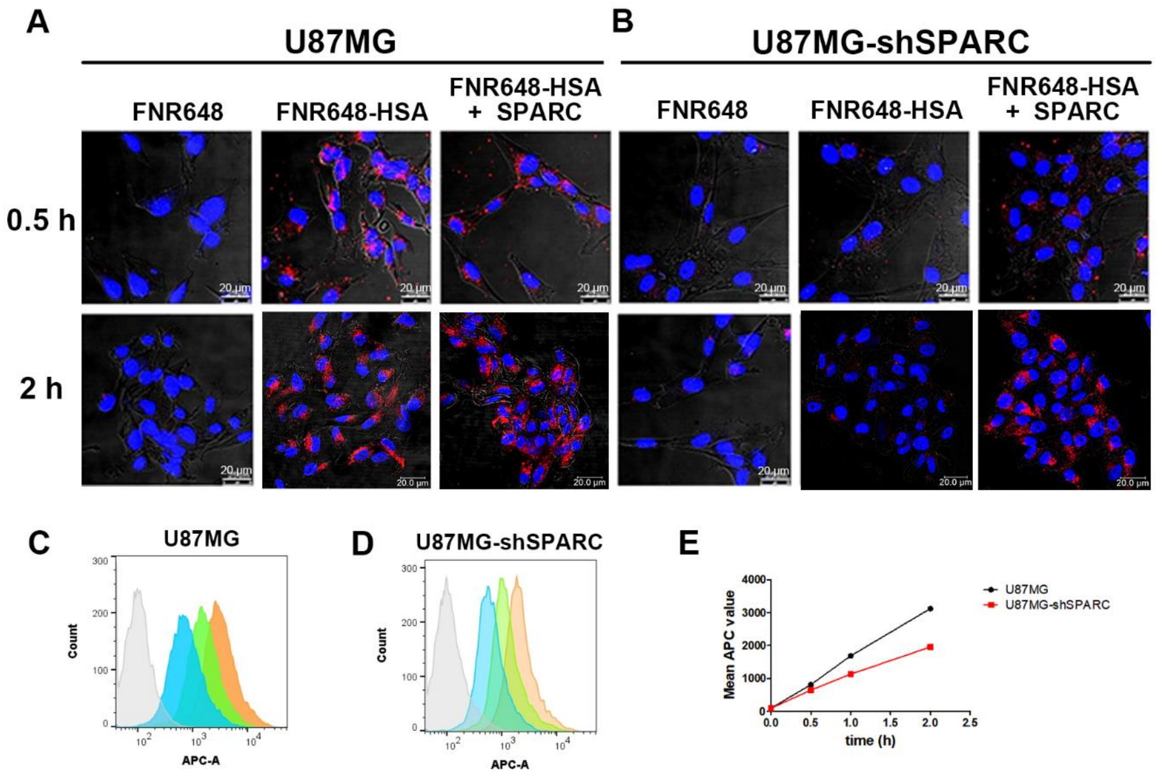

Figure S3. Cellular uptake of FNR648-HSA at different incubation time. Representative cell images of (A) U87MG and (B) U87MG-shSPARC cells. Scale bar, 20 μm. (C-E) Cellular uptake of FNR648-HSA in the course of time (0.5, 1, 2 h) from FACS data (C) U87MG and (D) U87MG-shSPARC APC histogram and (E) time versus APC mean intensity graph from FACS data.

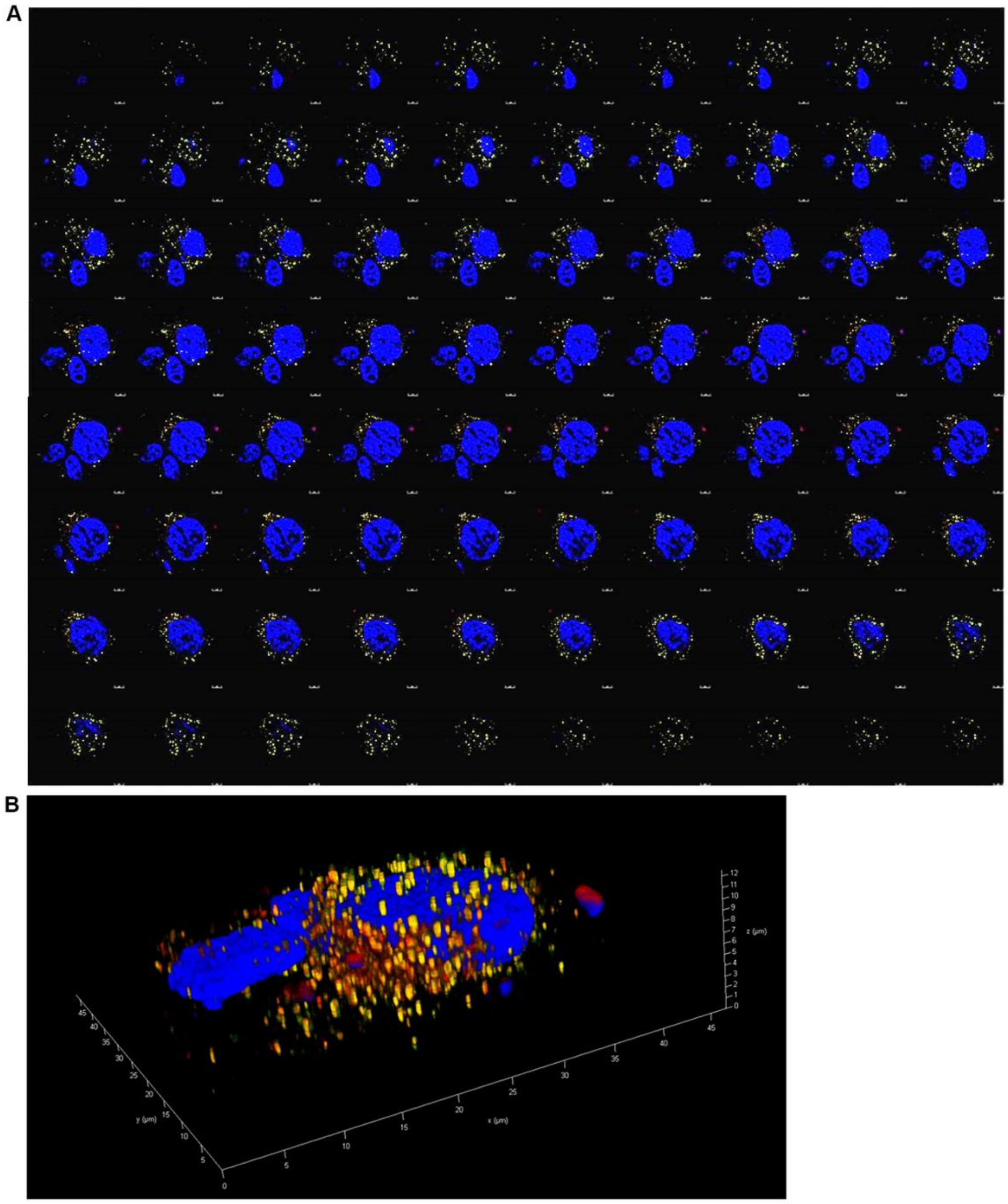

Figure S4. FRET analysis of Z stack images in U87MG cells. (A) Z stack and (B) 3D images. Total Z axis range was 8.4 µm, interval of each z-stack was 0.08 µm, and a total of 100 images were used for 3D images. Scale bar in (A), 5 µm. DAPI is shown in blue, Cy3-SPARC in green, and FNR648-HSA in red.

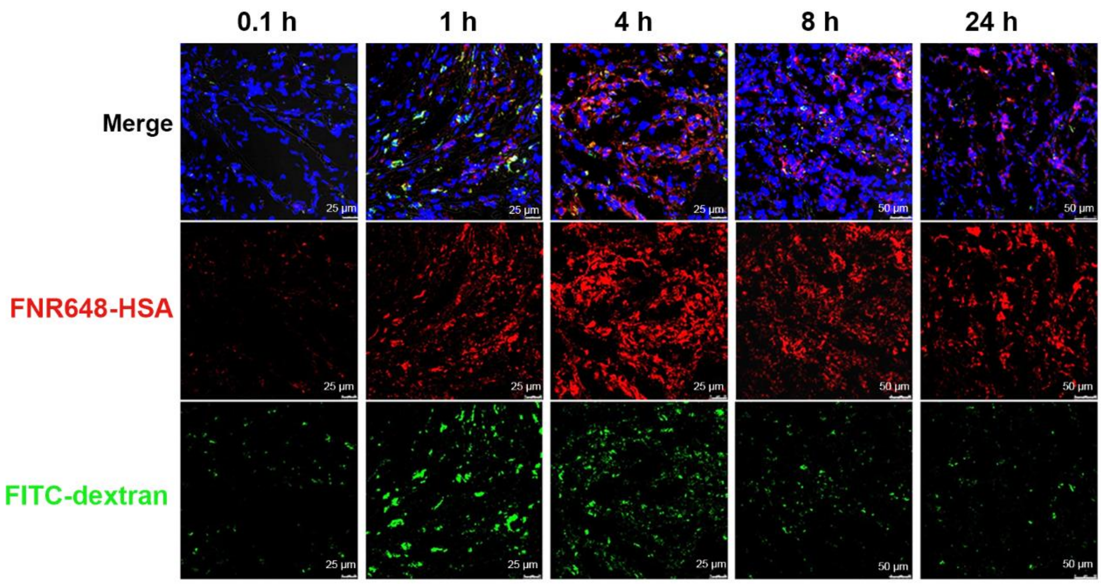

Figure S5. Representative micro-distribution of FNR648-HSA and FITC-dextran images in tumors. FNR648-HSA and FITC-dextran were imaged using confocal microscopy. Scale bar in (B), 25 μm and 50 μm. DAPI is shown in blue, FITC-dextran in green, and FNR648-HSA in red.

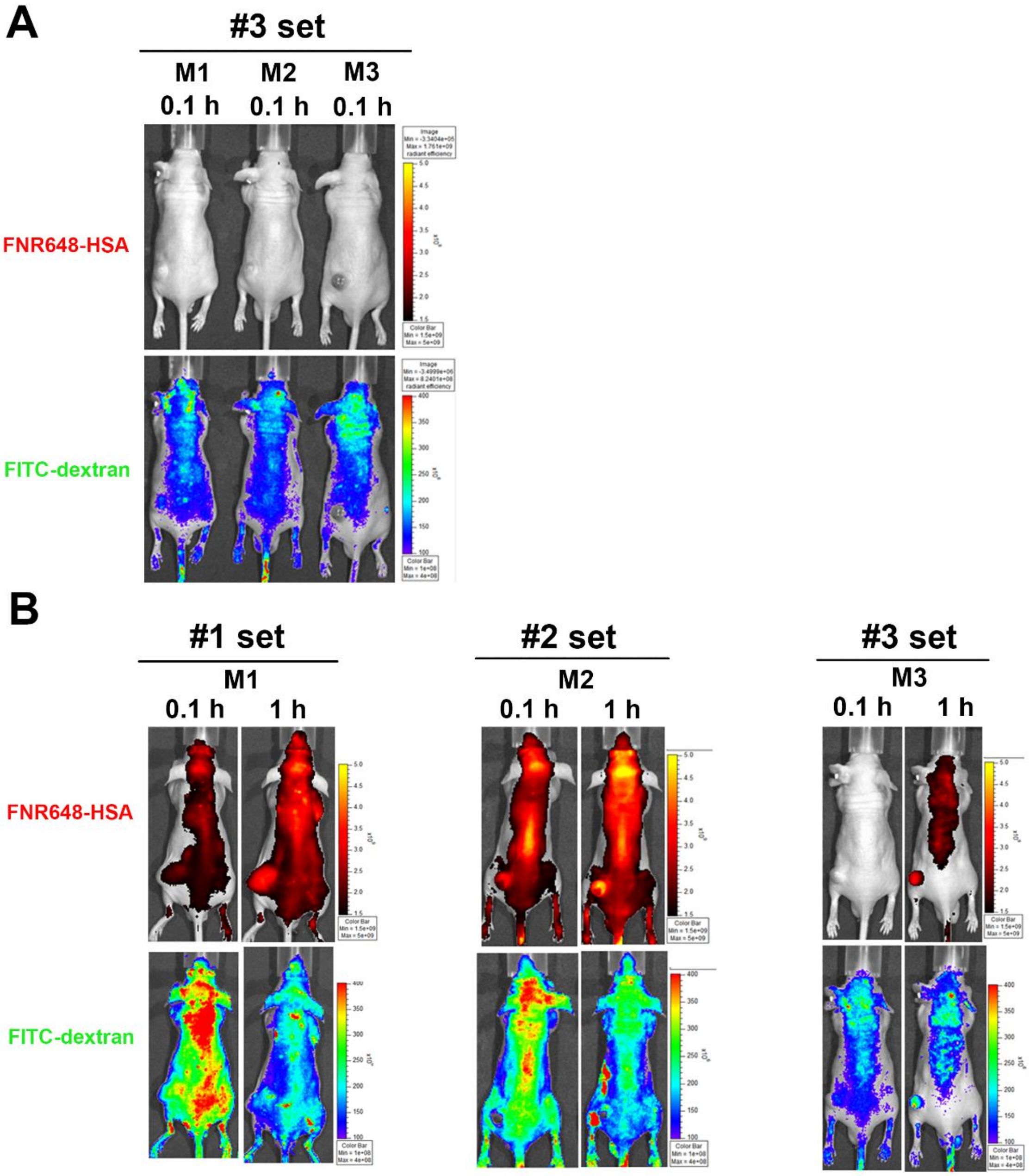

A
#3 set
M1
0.1 h
M2
0.1 h
M3
0.1 h
FNR648-HSA
FITC-dextran
B
#1 set
M1
0.1 h
1 h
#2 set
M2
0.1 h
1 h
#3 set
M3
0.1 h
1 h
FNR648-HSA
FITC-dextran

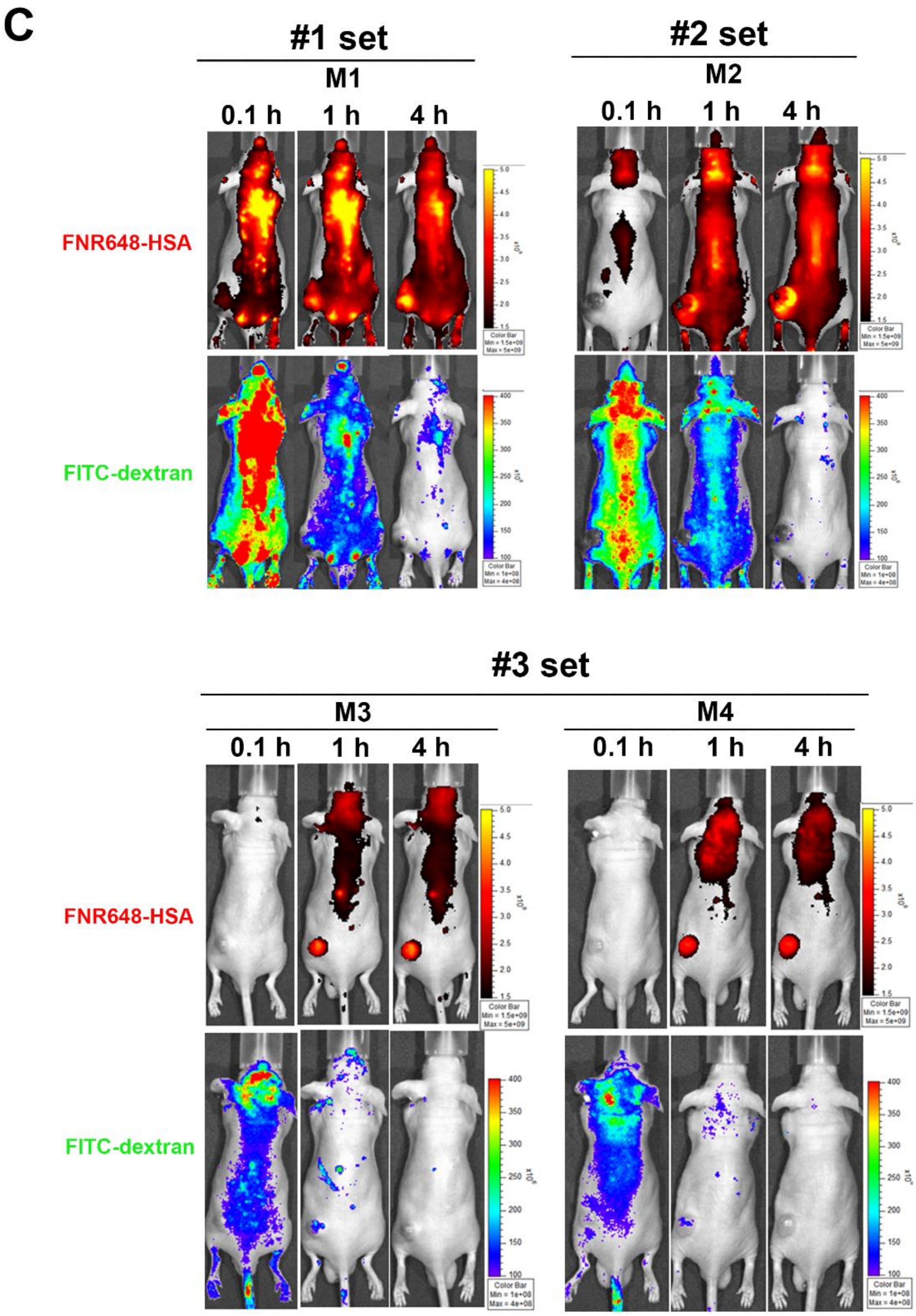
C
#1 set
M1
0.1 h
1 h
4 h
#2 set
M2
0.1 h
1 h
4 h
FNR648-HSA
FITC-dextran
#3 set
M3
0.1 h
1 h
4 h
M4
0.1 h
1 h
4 h
FNR648-HSA
FITC-dextran

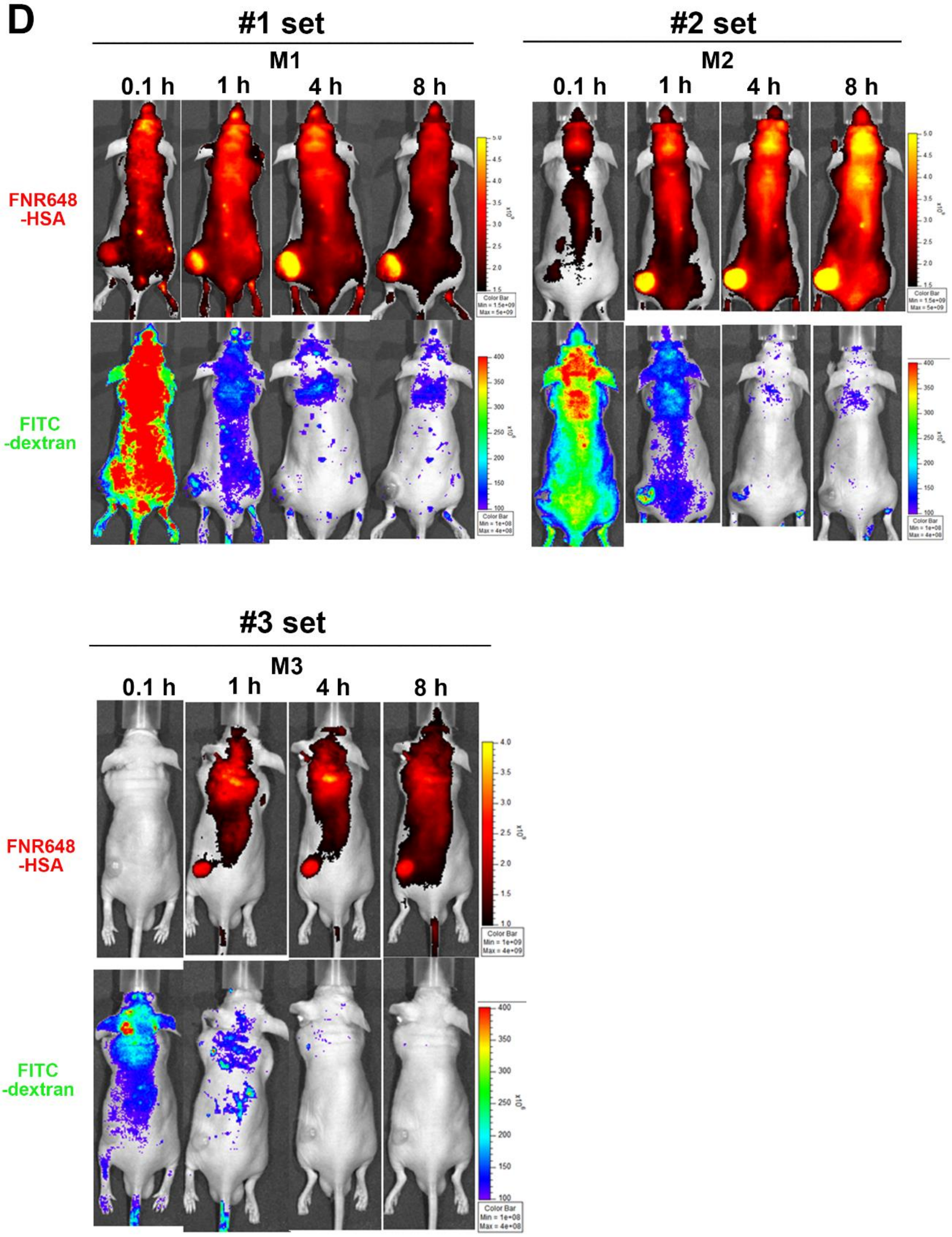
D
#1 set
M1
0.1 h
1 h
4 h
8 h
#2 set
M2
0.1 h
1 h
4 h
8 h
FNR648
-HSA
FITC
-dextran
#3 set
M3
0.1 h
1 h
4 h
8 h
FNR648
-HSA
FITC
-dextran

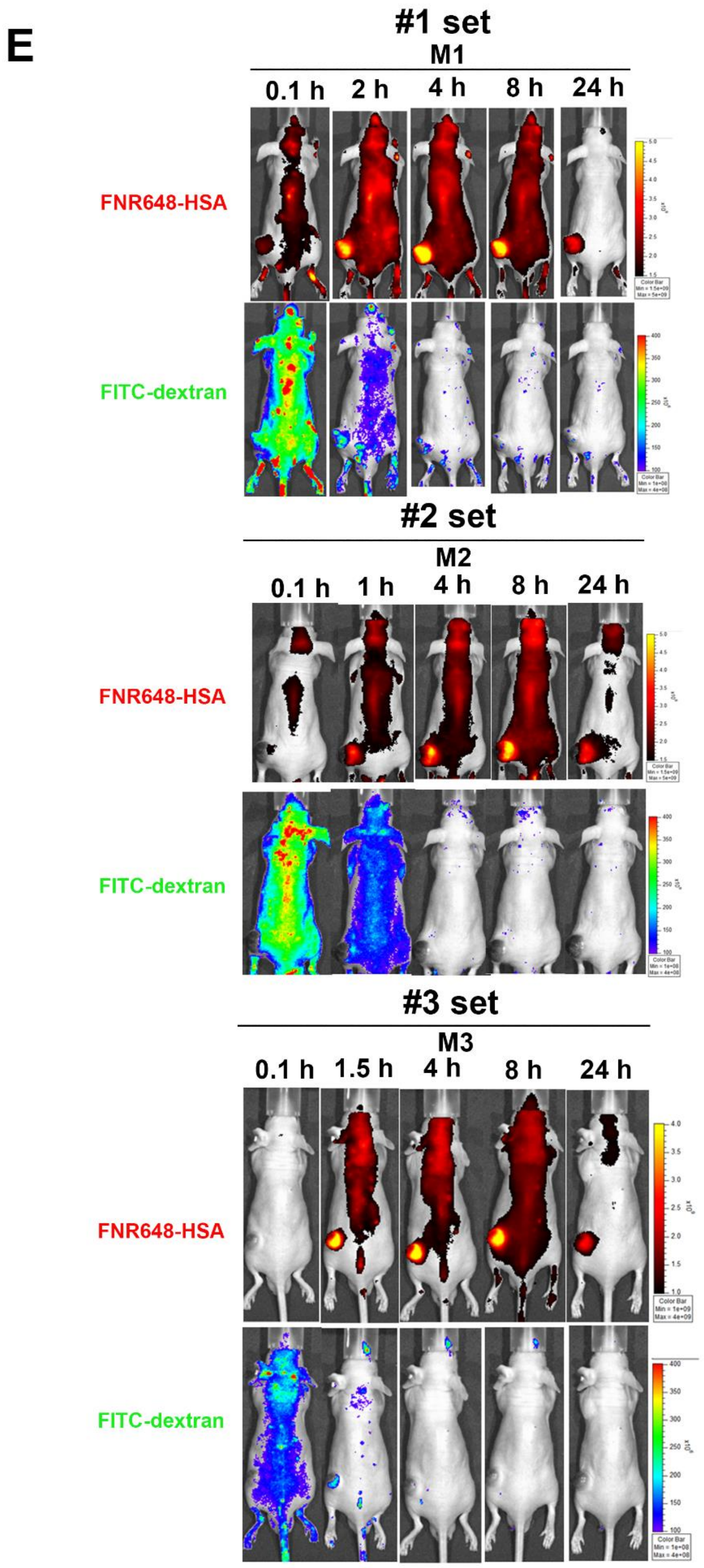
E
#1 set
M1
0.1 h
2 h
4 h
8 h
24 h
FNR648-HSA
FITC-dextran
#2 set
M2
0.1 h
1 h
4 h
8 h
24 h
FNR648-HSA
FITC-dextran
#3 set
M3
0.1 h
1.5 h
4 h
8 h
24 h
FNR648-HSA
FITC-dextran

Figure S6. *In vivo* images of mice acquired over time before sacrificing mice for tumor preparation. Time course images were acquired at (A) 0.1 h, (B) 1 h, (C) 4 h, (D) 8 h and (E) 24 h after intravenous injection of FNR648-HSA and FITC-dextran. Set means independent experiment and M number (M1, M2, M3) means mouse number at certain time point of group (n = 3 for 0.1, 1, 8, 24 h after injection and n = 4 for 4 h). Because of the different exposure time for acquiring images in each experiment, each experimental set is labeled. #1 and #2 set images were acquired using 2 sec exposure time, and #3 set images were acquired 1 sec exposure time.

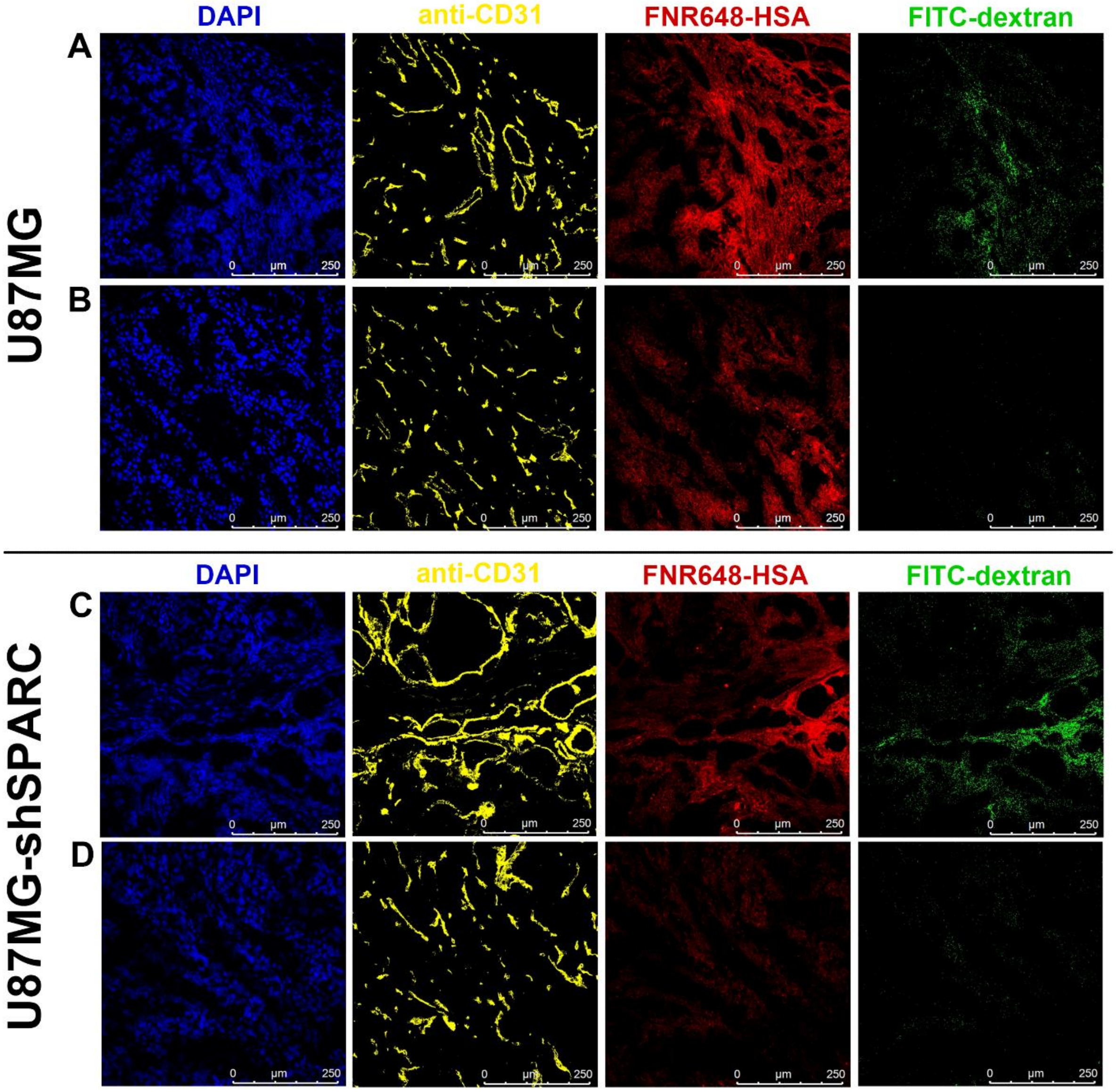

Figure S7. Micro-distribution of FNR648-HSA and FITC-dextran in tumor tissues. Separate images for each fluorescence signal from figure 5. (A, B) U87MG tumor region from Figure 5C and D. A is the same region as in Figure 5C, and B represents Figure 5D. (C, D) U87MG-shSPARC tumor region from Figure 5E and F. C is the same region as in Figure 5E, and D is Figure 5F. Scale bar, 250 μm.

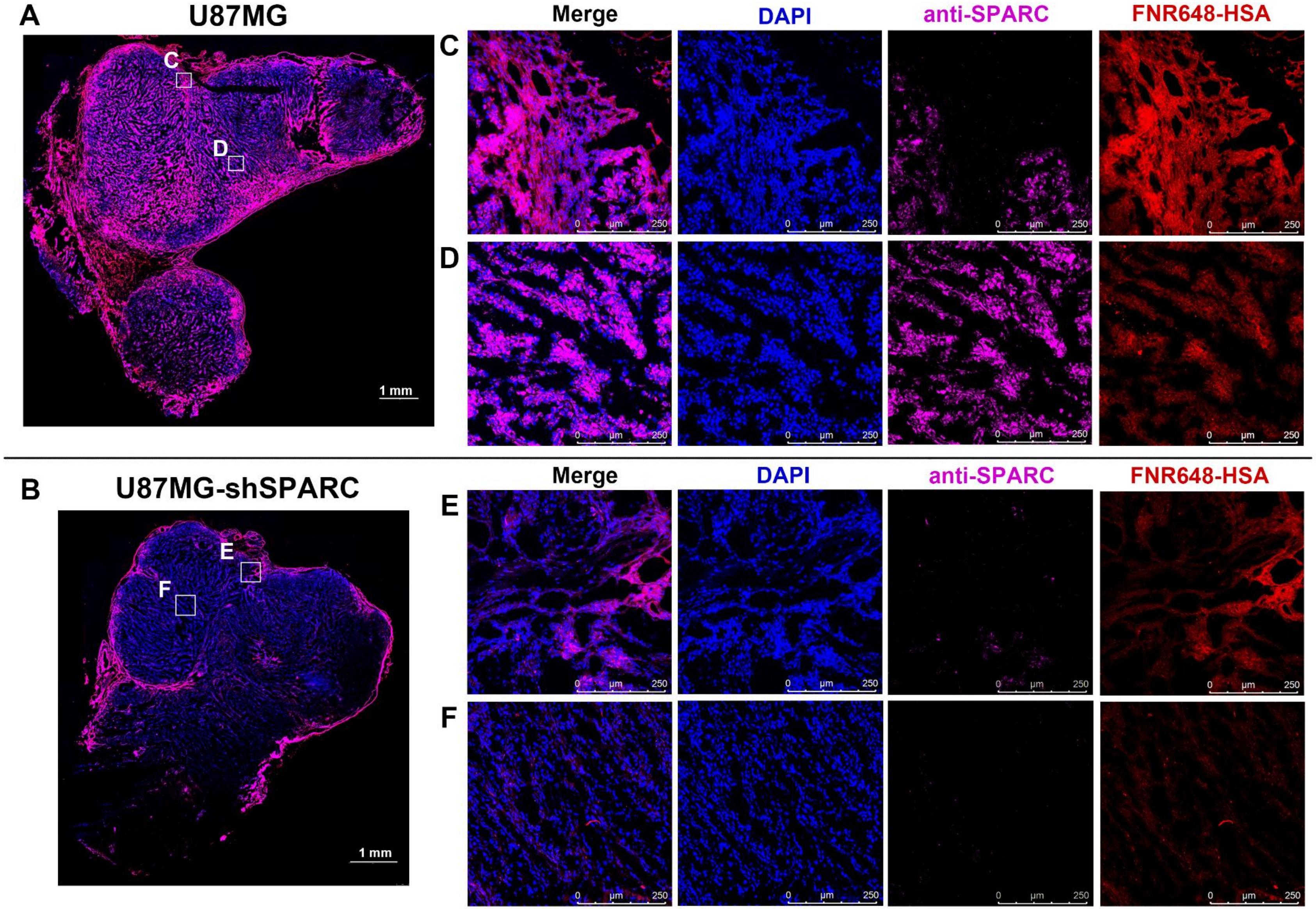

A
U87MG
C
D
1 mm
Merge
DAPI
anti-SPARC
FNR648-HSA
C
D
0 μm 250
B
U87MG-shSPARC
E
F
1 mm
Merge
DAPI
anti-SPARC
FNR648-HSA
E
F

Figure S8. Micro-distribution of SPARC in tumors. Whole tumor immunofluorescence images of (A) U87MGB and (B) U87MG-shSPARC. SPARC images were stained by anti-human SPARC antibody, and cell nuclei were stained by DAPI. White squares represent enlarged regions for each image (C, D, E, F for each image). Each image is labeled with its own scale bar, 1 mm or 250 μm.

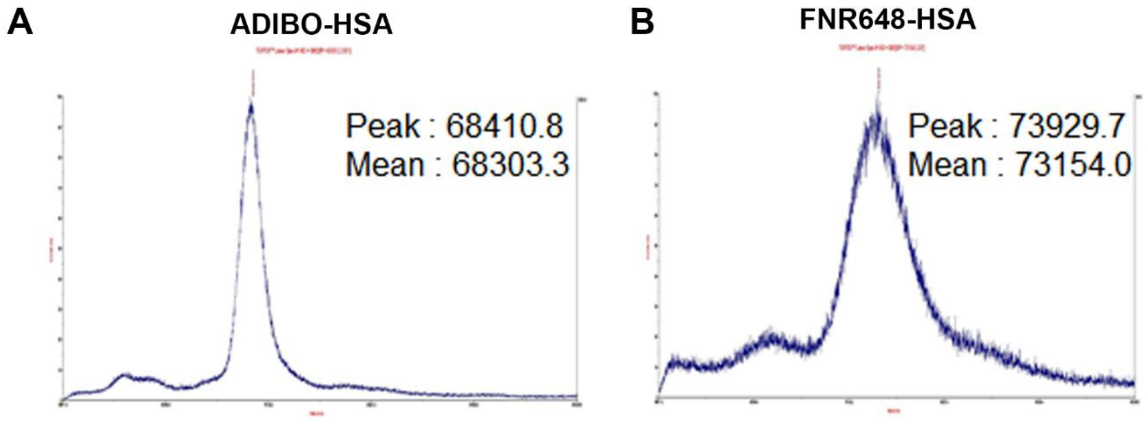

Figure S9. MALDI-TOF data of ADIBO-HSA and FNR648-HSA.

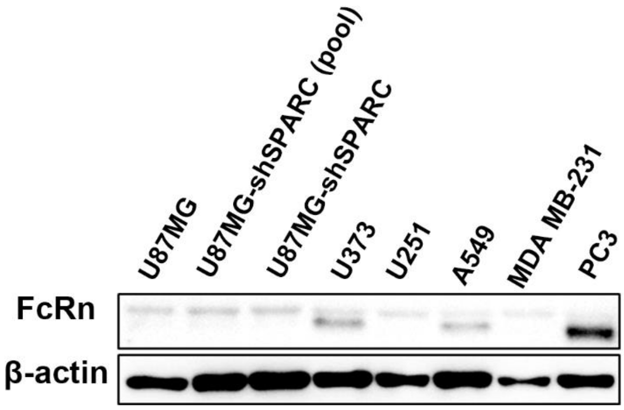

Figure S10. FcRn expression level in various cancer cells. FcRn protein expression levels were examined using anti-FcRn antibody.
